# A simple detection method for the serum sFLT1 protein in preeclampsia

**DOI:** 10.1038/s41598-021-00152-6

**Published:** 2021-10-18

**Authors:** Masabumi Shibuya, Haruka Matsui, Tadashi Sasagawa, Takeshi Nagamatsu

**Affiliations:** 1 Institute of Physiology and Medicine, Jobu University, Takasaki, Gunma Japan; 2 Department of Obstetrics and Gynecology, Faculty of Medicine, The University of Tokyo, Tokyo, Japan

**Keywords:** Biological techniques, Molecular biology, Biomarkers

## Abstract

In normal pregnancy, the soluble form of FMS-like tyrosine kinase-1 (sFLT1)/ vascular endothelial growth factor receptor-1 (sVEGFR-1), a VEGF-trapping protein, is expressed in trophoblasts of the placenta, suggesting that it plays an important role in the physiological barrier between fetal and maternal angiogenesis, when stimulated with VEGF-A. In pathological conditions such as preeclampsia (PE), sFLT1 protein is abnormally overexpressed in trophoblasts and secreted into the serum, which could cause hypertension and proteinuria on the maternal side and growth retardation on the fetal side. Detection of an abnormal increase in serum sFLT1 during the early to middle stages of PE is essential for proper initiation of medical care. To carry out this screening for sFLT1, we developed an easier and relatively low-cost sandwich-type ELISA method using a single mixture of human serum sample with an anti-FLT1 antibody and heparin-beads, namely heparin-beads-coupled ELISA (HB-ELISA). This method takes only about 2 h, and the sFLT1 values were similar levels with commercially available recent ELISA kits: the serum sFLT1 protein was approximately 4.3-fold increased in severe PE compared with those in normal pregnancy.

## Introduction

Preeclampsia (PE), a severe subtype of a hypertensive disorder of pregnancy (HDP), is a serious disease in the field of obstetrics, developing in approximately 5 to 7% of all pregnancies^1,2^. In 1990, we reported a new tyrosine kinase receptor carrying 7 immunoglobulin (Ig)-like domains in the extracellular region and named it FMS-like tyrosine kinase-1 (FLT1)^3^. Normal placental tissue was shown to express significant amounts of short mRNA (approximately 2–3 kb) in addition to the full-length FLT1 mRNA (approximately 8 kb long)^3^. FLT1 was found to tightly bind to vascular endothelial growth factor-A (VEGF-A), indicating that FLT1 is a receptor for VEGF, named as VEGFR-1^4,5^. In 1993, Kendall and Thomas reported that a short FLT1 mRNA encodes a VEGF-binding peptide that covers the 1 to 6 Ig-regions of the FLT1 with a 31 amino acid-long tail derived from intron-13, now known as i13 soluble FLT1 (i13 sFLT1)^5–7^. A low level of i13 sFLT1 is widely expressed in different types of cells in the body, and several groups have shown that the placental tissue expresses a high level of sFLT1^8,9^. In addition, another type of sFLT1, now called as e15a sFLT1, was found to be highly expressed in the placenta in primates, including humans^10–15^. Thus, trophoblasts express two major forms of sFLT1: i13, and e15a.

Structural differences between the two sFLT1 forms, sFLT1 i13 (687 amino-acids long) and e15a (734 amino acids long) are as follows: both proteins contain the same exon1-exon13 derived peptide of 656 amino acids long. This common region contains VEGF-A binding Immunoglobulin-like domain. At the carboxyl terminal region, i13 carries a short tail which is encoded in the intron 13. The e15a carries a carboxyl terminal tail different from i13, which is encoded in the exon 14 and exon 15a. The exon 15a is generated due to a non-physiological splicing of mRNA on the FLT1 gene.

From 2003 to 2004, serum sFLT1 protein levels were quantitatively examined and found to be abnormally expressed in PE patients^16–18^. The sFLT1 has a very strong VEGF-A trapping activity (Kd = 1–10 pM); thus, the two major symptoms of PE, hypertension and proteinuria, seem similar to the side effects frequently observed in cancer patients treated with a VEGF-A-neutralizing antibody such as bevacizumab^19^.

Within the human body, various types of cells such as vascular endothelial cells, macrophages in blood, podocytes in kidney, retinal neuronal cells, and trophoblasts were reported to express sFLT1^7,20,21^. Among these sFLT1-producing cells, trophoblasts in the placenta are unique in terms of higher expression of both i13 and e15a sFLT1 and lower expression of full-length FLT1^13^. sFLT1 is a very efficient VEGF-A/placenta growth factor (PlGF)/VEGF-B trapping molecule. Therefore, the biological significance of sFLT1 secreted from trophoblasts located between the fetal blood vessels and maternal vessels in the placenta appears to be an important biochemical barrier to suppress excess angiogenesis and vascular permeability in placental tissue^22^.

In PE patients, Levine et al. clearly showed that, at the early stage of pregnancy around 21 to 28 weeks of gestation, serum sFLT1 levels started to abnormally increase compared with those in normal pregnancy, even when clinical symptoms such as hypertension and proteinuria were not significant^18^.

These results suggest that earlier detection of sFLT1 increase in the serum could be a useful biomarker for any possible PEs in pregnancy. To date, sFLT1 detection ELISA (enzyme-linked immunosorbent assay) kits have been developed by various companies. However, these assays basically depend on two sFLT1-specific antibodies for sandwich-type ELISA method, and it takes about 5 h. The sFLT1/PlGF ratio in serum was reported to be a better biomarker than sFLT1 alone for PE^23–25^; however, for this assay, two different target-oriented ELISA systems are required. Here, we attempted to develop a simpler sFLT1 detection method for wide-scale screening of PE in the clinical setting, namely heparin-beads-coupled ELISA (HB-ELISA).

## Results and discussion

### Significant upregulation of sFLT1 in the serum of severe PE cases detected with new ELISA assay

**Figure 1:**
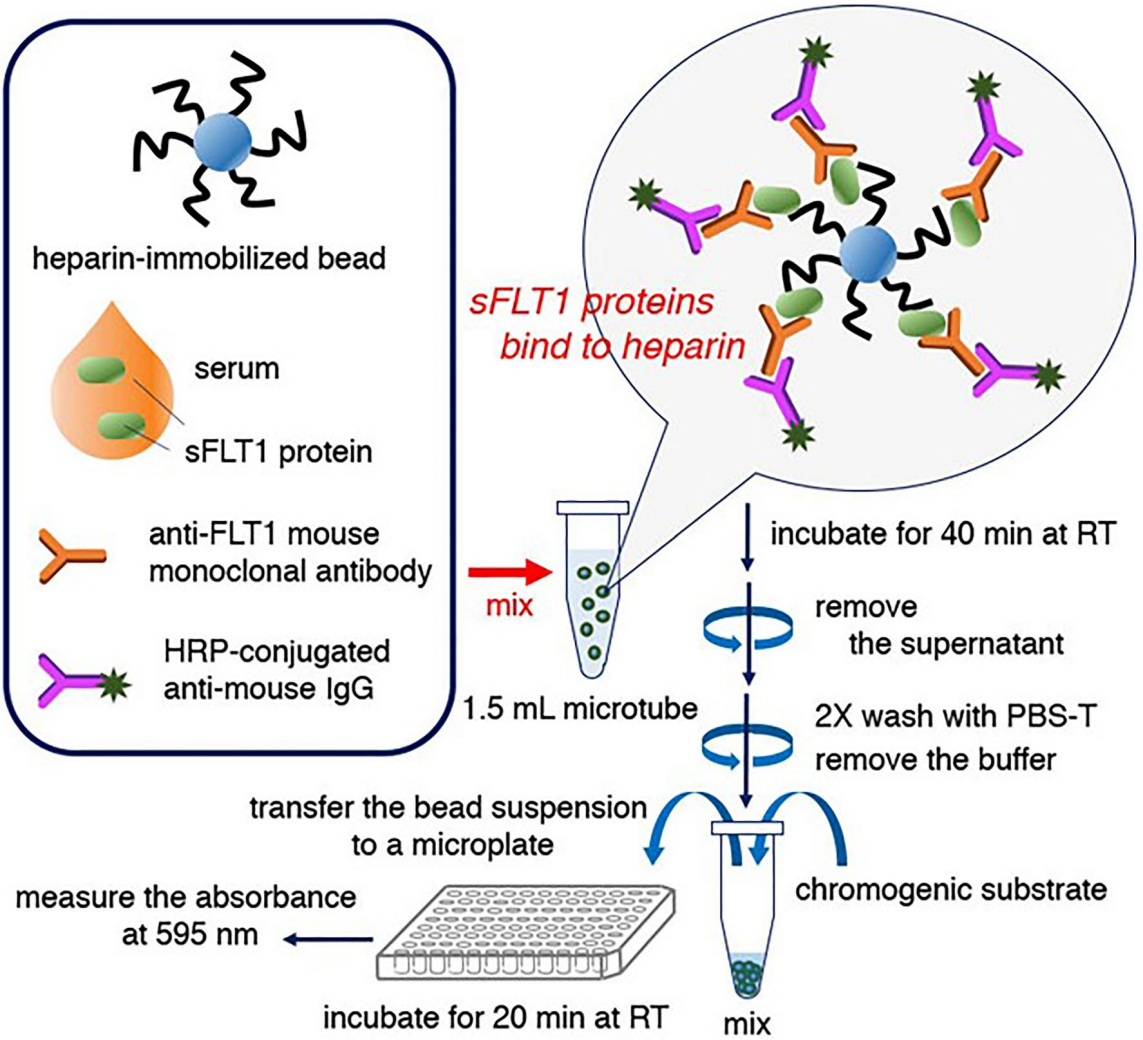
Schematic model for the new ELISA, heparin-beads-coupled ELISA (HB-ELISA).

**Figure 2:**
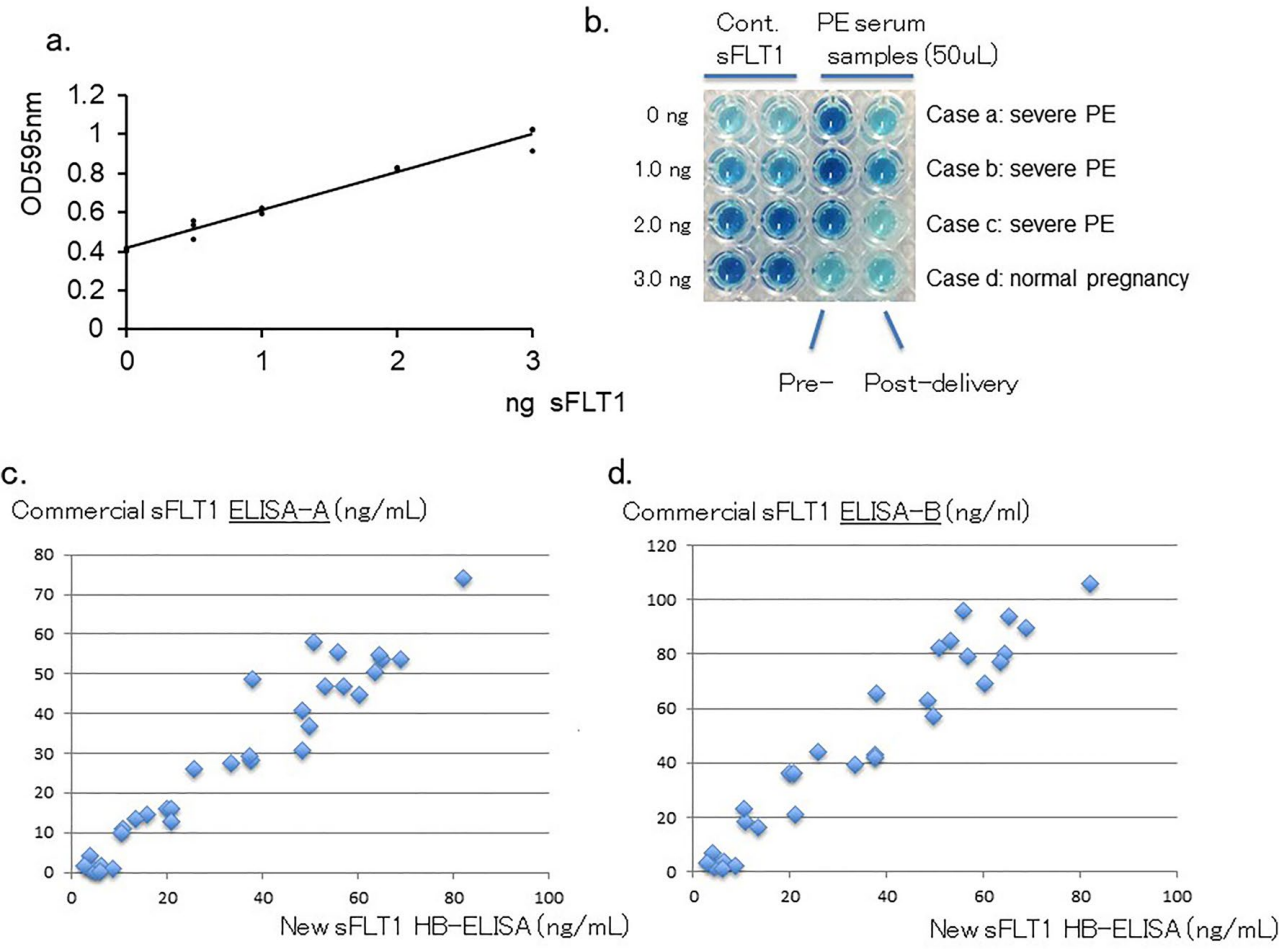
New sFLT1 assay system HB-ELISA detects similar levels of sFLT1 with those detected by commercially available recent sFLT1 ELISA kits. (**a**) A standard curve for sFLT1 assay. 0.5, 1.0, 2.0 and 3.0 soluble FLT1 (7 N FLT1) were assayed with this HB-ELISA three times for each. (**b**) a HB-ELISA assay for the serum samples from 3 severe-PE patients and one normal pregnancy. (**c**, **d**) Comparison of the sFLT1 values between the HB-ELISA and two commercially available recent ELISA kits. The sFLT1 values in the vertical axis were detected with commercial sFLT1 ELISA-A (**c**), and with ELISA-B (**d**). sFLT1 values in the horizontal axis (**c**, **d**) were detected with sFLT1 new HB-ELISA.

The sFLT1 proteins are known to tightly bind with heparin, and heparin-conjugated column is used to semi-purify those proteins from serum or protein-mixed solution. Therefore, we attempted to use heparin-immobilized beads (heparin-beads) and single sFLT1-specific antibody instead of two different sFLT1-specific antibodies for ELISA (Fig. 1). This new HB-ELISA showed stable values from 0.5 to 3.0 ng of purified sFLT1 in 1 mL-reaction mixture (Fig. 2a), and detected sFLT1 proteins in the 50 to 100uL serum samples of PE-patients (an example shown in Fig. 2b). A comparison of the sFLT1 values detected with the latest commercial ELISA kits and those from our assay showed that they were closely related to each other (Fig. 2c, d).

**Figure 3:**
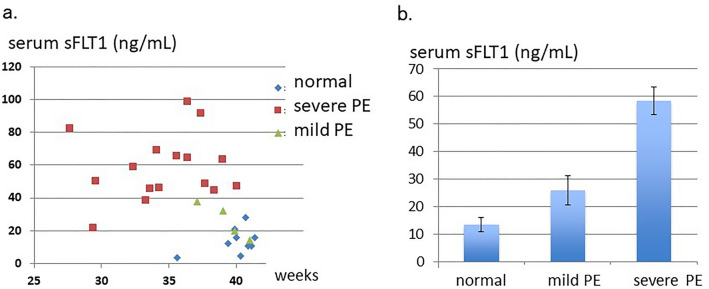
A significant increase in serum sFLT1 in severe PE cases. (**a**) sFLT1 levels (ng/mL) in serum samples detected with the HB-ELISA were shown with the time points when the serum sample was obtained (weeks: weeks of pregnancy). (**b**) The average values of sFLT1 in the serum samples from normal pregnancy, mild PE, and severe PE, are shown with SE. Increase in severe PE compared with the levels in normal pregnancy was statistically significant (p = 0.0016). The gestation weeks in the severe PE are 27w–5d to 40w–1d, the mild PE are 37w–1d to 41w–0d, and the normal pregnancy are 35w–4d to 41w–3d.

The distribution of sFLT1 values detected with this HB-ELISA during gestational weeks is shown in Fig. 3a. The average value of sFLT1 in 9 cases with normal pregnancy was 13.6 (SE ± 2.6) ng/mL serum, whereas the average of 20 PE cases showed 51.9 ± 5.1 ng/mL sFLT1 in serum, 3.8-fold higher than in normal pregnancy (p < 0.05). Among these PEs, 16 severe PE cases showed higher levels of sFLT1, 58.3 ± 5.0 ng/mL serum, which was 4.3-fold higher than those in normal cases (p < 0.05). On the other hand, 4 mild type PE cases showed only 26.0 ng/mL sFLT1 in serum, which was approximately a 1.9-fold increase, compared with those in normal pregnancy (Fig. 3b).

### A possible high production of sFLT1 in PE-placental tissues

The placental tissue gradually grows during pregnancy, and at an earlier stage of pregnancy, its weight and volume are lower. Thus, we attempted to revise the sFLT1 serum levels at the early phase of pregnancy, that is, possible sFLT1 value at the same weight of placental tissue. In general, the placental weight at full-term pregnancy is known to be 500–600 g, and during the weeks 25–30 of gestation, the weight of placenta is known to be about 250–300 g. In this study, the placental weight in one PE case delivered at 29 weeks and 6 days (29w–6d) was 290 g, and in 4 PE cases, the placental weights delivered during 30w–0d to 34w–6d were, on average, 405 g. The placentas in 8 PE cases with delivery between 35 and 41w were at an average of 518 g. Based on these results, we expected that the weight of the placenta from 25w–0d to 29w–6d was about 50% of that of a full-term placenta, and those during 30w–0d to 34w–6d were at 80% of the full-term placenta. Thus, the serum sFLT1 value in one PE case at 25w–0d to 29w–6d was divided by 0.5, and that from two PE cases from 30w–0d to 34w–6d was divided by 0.8 to adjust the sFLT1 value at a similar weight of placenta at full term. After this revision, sFLT1 levels in 16 cases with severe PE increased to 72.0 ± 7.8 ng/mL (p < 0.05) on average, approximately 5.3-fold higher than those in normal pregnancy.

However, the sizes of the placenta as well as the amounts of trophoblasts after 4-week gestation are variable among the pregnancy. Therefore, to obtain more accurate value for the production of sFLT1 from each placenta, we need more clinical data for these factors such as placental size and the ratio of trophoblasts and other types of cells.

### A comparison of the sFLT1 values detected with HB-ELISA and other ELISAs

This new assay method, HB-ELISA, takes only about 2 h at room temperature; thus, it appears simpler than other methods reported. The basal levels of sFLT1 in normal pregnancy, an average of 13.6 ng/mL in the serum detected in this new assay, were approximately threefold higher than those previously reported by others in 2003–2004^16–18^. The reason for this difference is not clear, but might be at least partly due to the use of heparin-beads at one side in our assay, which efficiently binds various sites of sFLT1. We previously showed that an artificially shortened sFLT1 peptide, which contains the first to third Ig domains of FLT1, about half of regular sFLT1, still efficiently bound with heparin-bead columns^26^. Thus, partly degraded sFLT1 peptide as well as an intact sFLT1 in the serum could be detectable in this assay.

**Figure 4:**
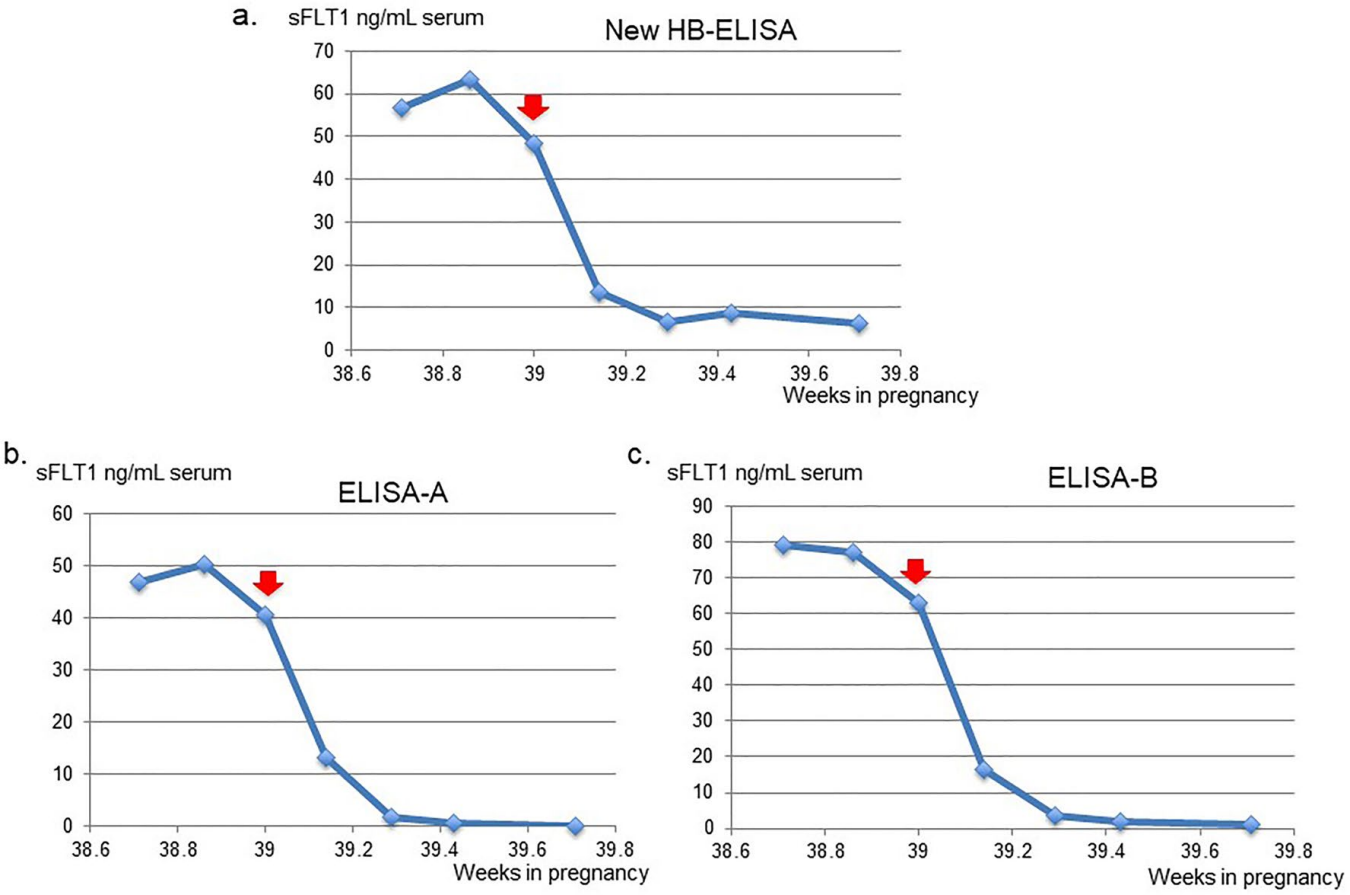
A rapid decrease in the serum sFLT1 levels after delivery in severe PE case. We compared the sFLT1 values detected with new HB-ELISA (**a**) and those with commercially available two sFLT1 ELISA kits, ELISA-A (**b**) and ELISA-B (**c**), using the same serum samples obtained from a single PE patient at 7 different time points. (**a**). A case of severe PE (PE-A) showed high sFLT1 levels, at approximately 60 ng/mL in the serum before delivery. Three days after delivery, the sFLT1 levels dramatically decreased to lower than 10. Red arrow: delivery date. “weeks”: weeks of pregnancy. (**b**, **c**) The sFLT1 values detected with two commercially available ELISA kits showed very similar sFLT1 patterns as that with new HB-ELISA method shown in (**a**).

In the severe PE cases with multiple time-course samples, high levels of sFLT1 before delivery, approximately 50–60 ng/mL, sharply decreased to very low levels, approximately 1/8–1/50, 5–9 days after delivery (Fig. 4a, Supplementary Fig. 1). In a case of PE, this pattern was confirmed with commercially available recent ELISA kits for sFLT1 (Fig. 4b, c). These results suggest that, after delivery of the fetus and placenta, the sFLT1 protein within the maternal blood is rapidly degraded and/or removed from the maternal body. Tang et al. reported that a significant amount of sFLT1 exists in the urine of pregnant women with PE, suggesting that one of the removal pathways of sFLT1 is via the kidney^25^.

The major merit of the HB-ELISA is a relatively simple and short-term sandwich-ELISA method. On the other hand, the sFLT1 values between 0 and 10 ng/mL in serum samples with this HB-ELISA appear to be a little higher compared with those detected with two antibody-used commercially available recent ELISA kits (Fig. 2c, d). These slight background values in HB-ELISA might be due to the use of a single antibody or heparin-beads. Practically, however, the HB-ELISA is sufficient for early detection of elevated sFLT1 in PE patients, which is higher than 20 ng/mL of sFLT1 in clinical serum samples.

### Other biomarkers in PE

Several groups have shown that sFLT1/PlGF ratio in serum is a good biomarker for PE^18,23–25^. Since the PlGF levels were found to decrease during the course of PE, this molar ratio, sFLT1/PlGF, is reasonable for the PE assay to be better than a single assay for sFLT1 or PlGF. However, the assay for determining this ratio of the two molecules might be time-consuming and of relatively high cost because of the requirement of four specific antibodies, two for sFLT1 and two for PlGF.

Several factors, including sFLT1 and sEndoglin upregulated by various stresses during pregnancy, have been considered to play important roles in the pathogenesis of PE^22,27–31^. Among these, various results of studies involving PE animal models and pilot studies with the removal of sFLT1 in the clinical field strongly support that sFLT1 is a major direct cause of hypertension and proteinuria in PE^16,32–38^. Therefore, a simple sFLT1 detection method described here, HB-ELISA, could be useful for early-stage screening of severe PE.

### Validation assay

**Figure 5:**
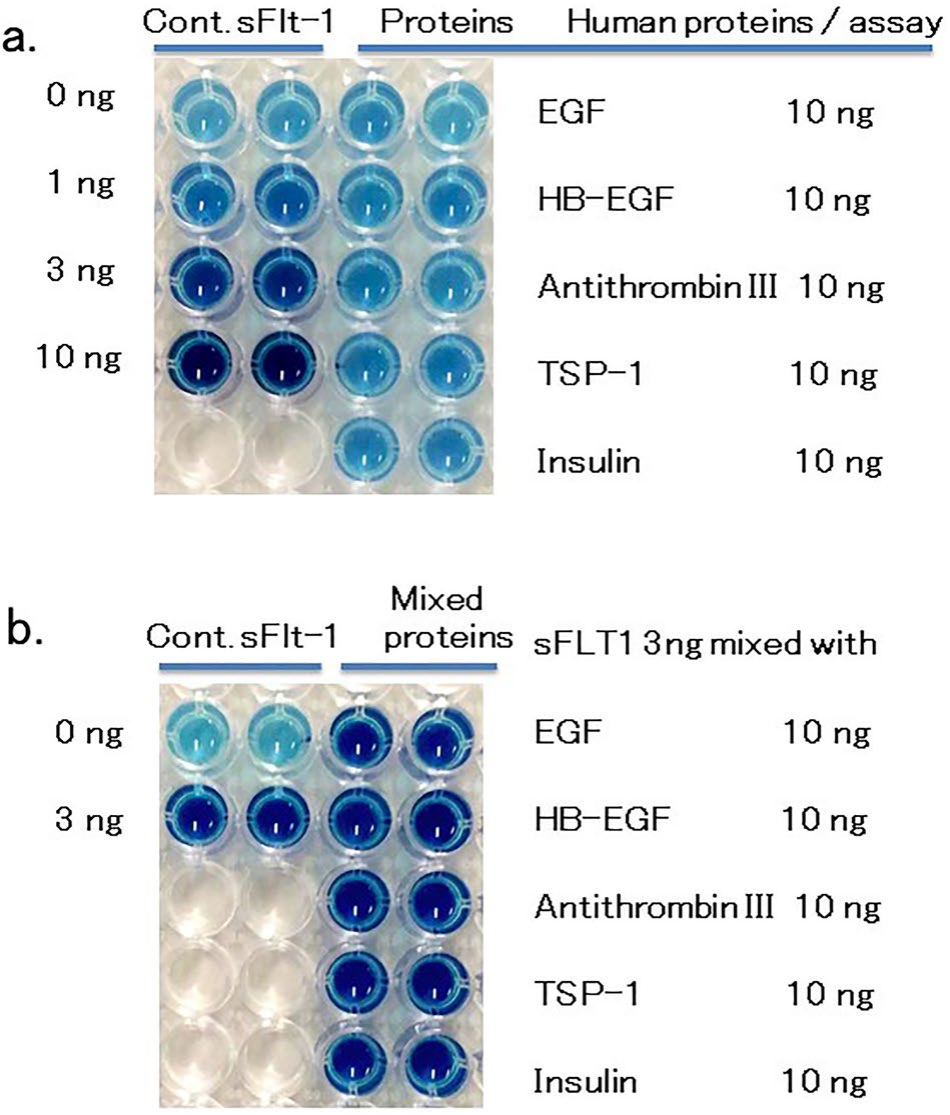
Validation assay was carried out with 5 human proteins: EGF, HB-EGF, Antithrombin III, TSP-1 and Insulin. (**a**) 10 ng of these proteins were assayed with sFLT1 HB-ELISA. The values detected were: 0 ± 0.55, 0.37 ± 0.31, 0.24 ± 0.18, 0.46 ± 0.17, and 0.23 ± 0.03, respectively. (**b**) 10 ng of these proteins were mixed with control 3 ng of sFLT1, and assayed with sFLT1 HB-ELISA. The values detected were: 2.88 ± 0.32, 3.19 ± 0.77, 2.98 ± 0.10, 3.08 ± 0.22, and 2.84 ± 0.08, respectively.

To further validate the sFLT1 HB-ELISA, we tested several human proteins with the HB-ELISA. Non-heparin-binding type: Insulin and Epidermal growth factor (EGF)^39^; and heparin-binding type: Thrompospondin-1 (TSP-1)^40^, Heparin-binding EGF-like growth factor (HB-EGF)^39^ and Antithrombin III^41^ were examined. As indicated in Fig. 5a, non of these proteins (10 ng) showed significant cross reaction with sFLT1 HB-ELISA. As a minor level, however, we detected a weak positive value with TSP-1 (about 0.5 value with the 10 ng of TSP-1; i.e., about 5% level of sFLT1). TSP-1 was reported to be about 207 ng/ml in the serum of normal pregnancy, and 11%-decrease in the severe PE (non PE: 207 ng/ml serum; severe PE: about 183 ng/ml)^40^. Since each serum sample for the sFLT1 assay contains basal level of TSP-1, and the difference in the TSP-1 between non PE and severe PE is not so high, we conclude that the sFLT1 values detected with this HB-ELISA are not significantly modulated with the TSP-1.

The concentration of the endogenous EGF and HB-EGF in the serum was reported to be much lower than sFLT1^39^ (lower than about 1 ng/mL for EGF, and 100 pg/mL for HB-EGF both in normal pregnancy and PE). Also, endogenous Insulin is known to be lower than sFLT1, about 0.1–0.4 ng/mL (3–10 uU/mL) in the blood.

We next examined whether any of these proteins have a suppressive effect on this HB-ELISA. To test this, we mixed 10 ng of each protein with 3 ng of control sFLT1, and assayed with this HB-ELISA. As shown in Fig. 5b, non of the proteins had significant suppressive effect on this HB-ELISA (detected values: 2.8–3.2 indicated in Fig. 5 legend). Taken together, these results strongly suggest that the newly developed simple sFLT1 assay, HB-ELISA, has a sufficient specificity for the screening of PE-candidate patients.

## Materials and methods

### Clinical samples

All procedures involving serum analysis were approved by the Ethical Committee of the Jobu University (No.17-H01; http://www.jobu.ac.jp/summary /pdf/h29_hito_approvalresult.pdf) and the institutional review board of the Faculty of Medicine, University of Tokyo (IRB number: 10580), respectively. After obtaining informed consent, the sera were collected from healthy pregnant women and those with PE. Approximately 0.5 mL serum per patient from among nine patients with normal pregnancy, four patients with mild PE, and 16 patients with severe PE were obtained pre- and/or post-delivery and stored at − 80 °C until use. PE was diagnosed following the clinical criteria defined by Japanese Society of Obstetrics and Gynecology in 2018: for mild PE, hypertension > 140/90 mmHg, and lower than 160/110 mmHg with proteinuria higher than 300 mg/day and lower than 2 g/day; for severe PE, hypertension > 160/110 mmHg and proteinuria > 2 g/day. Clinical samples such as blood plasma obtained with anti-coagulant heparin are not suitable for the assay.

### Materials

Heparin-immobilized beads (Heparin Sepharose: 6 Fast Flow, 17-0998-01) was purchased from GE Healthcare (Uppsala, Sweden). An anti sFLT1 mouse monoclonal antibody KM1750^42^, which recognizes the 2–3-Ig regions of the human FLT1, was used as the primary antibody. Horseradish peroxidase (HRP)-labeled anti-mouse immunoglobulin goat serum (ab6789, Abcam, Cambridge, UK) was used as the secondary antibody. HRP assay solution (Substrate Reagent Pack, DY999) was obtained from R&D Systems (Minneapolis, U.S.A.), and two sFLT1-ELISA kits were purchased from Invitrogen (California, U.S.A.) and R&D Systems.

### Methods

A sandwich ELISA system using heparin-beads (heparin-Sepharose) and an anti-sFLT1 antibody, i.e. heparin-beads-coupled ELISA (HB-ELISA) (Fig. 1), was constructed as follows; in 1.0 mL assay solution, 100 µL volume included human serum (clinical sample) or inactivated fetal bovine serum (FBS) (56 °C-treated for 30 min: negative control) or both. For a standard sFLT1 assay, we used 7N FLT1 protein (1–7 Ig domain-containing soluble FLT1), which was expressed in SF9-insect cells with a Baculo-virus vector and purified with a heparin-beads column and gel-filtration column^26^. 7N sFLT1 has a peptide 750 amino acids long, similar to 6 N i13 (688 amino acids) and e15a (734 amino acids) sFLT1. 7 N sFLT-1 protein (0.3, 1.0, or 3.0 ng) was mixed with 100 µL of inactivated FBS. Typically, 20 µL, 50 µL, or 100 µL of human sera clinical samples were assayed. When 20 µL or 50 µL of human serum was assayed, the total volume was adjusted to 100 µL by adding the inactivated FBS.

A total of 1.0 mL solution (basic solution: PBS-T; phosphate-buffered saline with 0.1% Tween20), which included (1) clinical serum sample, (2) heparin-beads (about 30 µL volume/assay), (3) anti-sFLT1 antibody (140 ng/assay), and (4) the anti-mouse monoclonal goat antibody labeled with HRP (200 ng/assay) in 1.5 mL-microtube, was gently mixed for 40 min at room temperature. After incubation, 1.5 mL-microtubes were centrifuged at 300×*g* for 8 s (s), twice, and the supernatant solutions were carefully removed from the heparin-beads-antibody precipitates. The precipitates were washed with 1 mL of PBS-T for 5 min at room temperature with gentle mixing, then centrifuged at 300×*g* for 8 s, twice. After removing the supernatant as much as possible, this washing step with 1 mL of PBS-T was repeated once more. The heparin-beads-based sFLT1 precipitates were added with 200 µL of HRP assay solution. The mixture of sFLT1-bound heparin-beads, anti-sFLT1 antibody and HRP-labeled anti-mouse Ig antibody with HRP assay-solution was transferred to 96-well plates, incubated for 20 min at room temperature, and then assayed with an iMark Microplate Reader (Bio-Rad Laboratories Inc., CA, USA) at 595 nm (Fig. 1).

### Statistical analysis

The data are expressed as the mean ± standard error (SE) and were analyzed by an unpaired *t*-test for parametric data. Statistical analyses were performed using Excel 2011 (Microsoft, Seattle, WA, USA) with the add-in software Statcel4 (OMS, Tokyo, Japan). A value of p < 0.05 was considered statistically significant.

### Validation experiments

For the validation of the HB-ELISA, we used several human proteins: for the non-heparin-binding proteins, Insulin (FUJIFILM Wako Pure Chemical Corporation, Osaka, Japan) and EGF (FUJIFILM Wako Pure Chemical Corporation, Osaka, Japan); and for the heparin-binding proteins, TSP-1 (R&D Systems, MN, USA), HB-EGF (R&D Systems, MN, USA), and Antithrombin III (Sigma-Aldrich, St. Louis, MO, USA).

### Experimental methods guideline statement

All experiments were performed in accordance with relevant guidelines and regulations.

## Supplementary Information

Supplementary Information.

## References

[1] Wang A, Rana S, Karumanchi SA (2009). Physiology (Bethesda)..

[2] Young BC, Levine RJ, Karumanchi SA (2010). Pathogenesis of preeclampsia. Annu. Rev. Pathol..

[3] Shibuya M (1990). Nucleotide sequence and expression of a novel human receptor-type tyrosine kinase gene (flt) closely related to the fms family. Oncogene.

[4] Ferrara N (2004). Vascular endothelial growth factor: Basic science and clinical progress. Endocr. Rev..

[5] Kendall RL, Thomas KA (1993). Inhibition of vascular endothelial cell growth factor activity by an endogenously encoded soluble receptor. Proc. Natl. Acad. Sci. USA.

[6] Kondo K, Hiratsuka S, Subbalakshmi E, Matsushime H, Shibuya M (1998). Genomic organization of the *flt-1* gene encoding for Vascular Endothelial Growth Factor (VEGF) Receptor-1 suggests an intimate evolutionary relationship between the 7-Ig and the 5-Ig tyrosine kinase receptors. Gene.

[7] Shibuya M (2006). Vascular endothelial growth factor receptor-1 (VEGFR1/Flt-1): A dual regulator for angiogenesis. Angiogenesis.

[8] Clark DE (1998). A vascular endothelial growth factor antagonist is produced by the human placenta and released into the maternal circulation. Biol. Reprod..

[9] He Y (1999). Alternative splicing of vascular endothelial growth factor (VEGF)-R1 (FLT-1) pre-mRNA is important for the regulation of VEGF activity. Mol. Endocrinol..

[10] Thomas CP, Andrews JI, Liu KZ (2007). Intronic polyadenylation signal sequences and alternate splicing generate human soluble Flt1 variants and regulate the abundance of soluble Flt1 in the placenta. FASEB J..

[11] Sela S (2008). A novel human-specific soluble vascular endothelial growth factor receptor 1: Cell-type-specific splicing and implications to vascular endothelial growth factor homeostasis and preeclampsia. Circ. Res..

[12] Heydarian M (2009). Novel splice variants of sFlt1 are upregulated in preeclampsia. Placenta.

[13] Jebbink J (2011). Expression of placental FLT1 transcript variants relates to both gestational hypertensive disease and fetal growth. Hypertension.

[14] Rajakumar A (2009). Novel soluble Flt-1 isoforms in plasma and cultured placental explants from normotensive pregnant and preeclamptic women. Placenta.

[15] Thomas CP (2009). A recently evolved novel trophoblast-enriched secreted form of fms-like tyrosine kinase-1 variant is up-regulated in hypoxia and preeclampsia. J. Clin. Endocrinol. Metab..

[16] Maynard SE (2003). Excess placental soluble fms-like tyrosine kinase 1 (sFlt1) may contribute to endothelial dysfunction, hypertension, and proteinuria in preeclampsia. J. Clin. Invest..

[17] Koga K (2003). Elevated serum soluble vascular endothelial growth factor receptor 1 (sVEGFR-1) levels in women with preeclampsia. J. Clin. Endocrinol. Metab..

[18] Levine RJ (2004). Circulating angiogenic factors and the risk of preeclampsia. N. Engl. J. Med..

[19] Hurwitz H (2004). Bevacizumab plus irinotecan, fluorouracil, and leucovorin for metastatic colorectal cancer. N. Engl. J. Med..

[20] Jin J (2012). Soluble FLT1 binds lipid microdomains in podocytes to control cell morphology and glomerular barrier function. Cell.

[21] Luo L (2013). Photoreceptor avascular privilege is shielded by soluble VEGF receptor-1. Elife.

[22] Shibuya M (2011). Involvement of Flt-1 (VEGF receptor-1) in cancer and preeclampsia. Proc. Jpn. Acad. Ser. B Phys. Biol. Sci..

[23] Doherty A (2014). Altered hemodynamics and hyperuricemia accompany an elevated sFlt-1/PlGF ratio before the onset of early severe preeclampsia. J. Obstet. Gynaecol. Can..

[24] Hund M (2015). Influence of the sFlt-1/PlGF ratio on clinical decision-making in women with suspected preeclampsia—The PreOS study protocol. Hypertens. Pregnancy..

[25] Tang P (2017). Use of serum and urinary soluble sFlt-1 and PLGF in the diagnosis of preeclampsia. Hypertens. Pregnancy.

[26] Tanaka K, Yamaguchi S, Sawano A, Shibuya M (1997). Characterization of the extracellular domain in vascular endothelial growth factor receptor-1 (Flt-1 tyrosine kinase). Jpn. J. Cancer Res..

[27] Levine RJ (2006). Soluble endoglin and other circulating antiangiogenic factors in preeclampsia. N. Engl. J. Med..

[28] Foidart JM, Schaaps JP, Chantraine F, Munaut C, Lorquet S (2009). Dysregulation of anti-angiogenic agents (sFlt-1, PLGF, and sEndoglin) in preeclampsia–a step forward but not the definitive answer. J. Reprod. Immunol..

[29] Munaut C (2008). Hypoxia is responsible for soluble vascular endothelial growth factor receptor-1 (VEGFR-1) but not for soluble endoglin induction in villous trophoblast. Hum. Reprod..

[30] Sasagawa T (2018). HIF-2α, but not HIF-1α, mediates hypoxia-induced up-regulation of Flt-1 gene expression in placental trophoblasts. Sci. Rep..

[31] Nagamatsu T (2004). Cytotrophoblasts up-regulate soluble fms-like tyrosine kinase-1 expression under reduced oxygen: An implication for the placental vascular development and the pathophysiology of preeclampsia. Endocrinology.

[32] Lu F (2007). The effect of over-expression of sFlt-1 on blood pressure and the occurrence of other manifestations of preeclampsia in unrestrained conscious pregnant mice. Am. J. Obstet. Gynecol..

[33] Thadhani R (2011). Pilot study of extracorporeal removal of soluble fms-like tyrosine kinase 1 in preeclampsia. Circulation.

[34] Nakakita B (2015). Case of soluble fms-like tyrosine kinase 1 apheresis in severe pre-eclampsia developed at 15 weeks' gestation. J. Obstet. Gynaecol. Res..

[35] Thadhani R (2016). Removal of soluble Fms-Like tyrosine kinase-1 by dextran sulfate apheresis in preeclampsia. J. Am. Soc. Nephrol..

[36] Shibuya M (2013). Vascular endothelial growth factor and its receptor system: Physiological functions in angiogenesis and pathological roles in various diseases. J. Biochem..

[37] Contini C (2018). Lipoprotein turnover and possible remnant accumulation in preeclampsia: Insights from the Freiburg Preeclampsia H.E.L.P.-apheresis study. Lipids Health Dis..

[38] Winkler K (2018). Treatment of very preterm preeclampsia via heparin-mediated extracorporeal LDL-precipitation (H.E.L.P.) apheresis: The Freiburg preeclampsia H.E.L.P.-Apheresis study. Pregnancy Hypertens..

[39] Armant DR (2015). Reduced expression of the epidermal growth factor signaling system in preeclampsia. Placenta.

[40] Ulu I (2019). Maternal serum thrombospondin-1 is significantly altered in cases with established preeclampsia. Matern. Fetal Neonatal Med..

[41] Sarkar PD, Sogani S (2013). Association of antithrombin-III and platelet count with pregnancy induced hypertension. Int. J. Reprod. Contracept. Obstet. Gynecol..

[42] Kanno S (2000). Roles of two VEGF receptors, Flt-1 and KDR, in the signal transduction of VEGF effects in human vascular endothelial cells. Oncogene.

