# Supplementary material for: A simple detection method for the serum sFLT1 protein in preeclampsia
Source: Sci Rep. 2021 Oct 18;11:20613. doi: 10.1038/s41598-021-00152-6 (PMC8523687; doi:10.1038/s41598-021-00152-6)
Supplement: Supplementary file 1 — Supplementary Information. [file 41598_2021_152_MOESM1_ESM.pdf]

A simple detection method for the serum sFLT1 protein in preeclampsia.

Masabumi Shibuya[1,*], Haruka Matsui[2], Tadashi Sasagawa[1] & Takeshi Nagamatsu[2]

[1]Institute of Physiology and Medicine, Jobu University, Gunma, Japan.
[2]Department of Obstetrics and Gynecology, Faculty of Medicine, The University of Tokyo, Tokyo, Japan.
*Correspondence

Tel: +81 274-20-2115
Fax: +81 274-42-5204
E-mail: shibuya@ims.u-tokyo.ac.jp

Keywords: sFLT1, preeclampsia, simple detection.

Running title: A simple detection method for serum sFLT1

Supplementary Figure 1. (Shibuya)

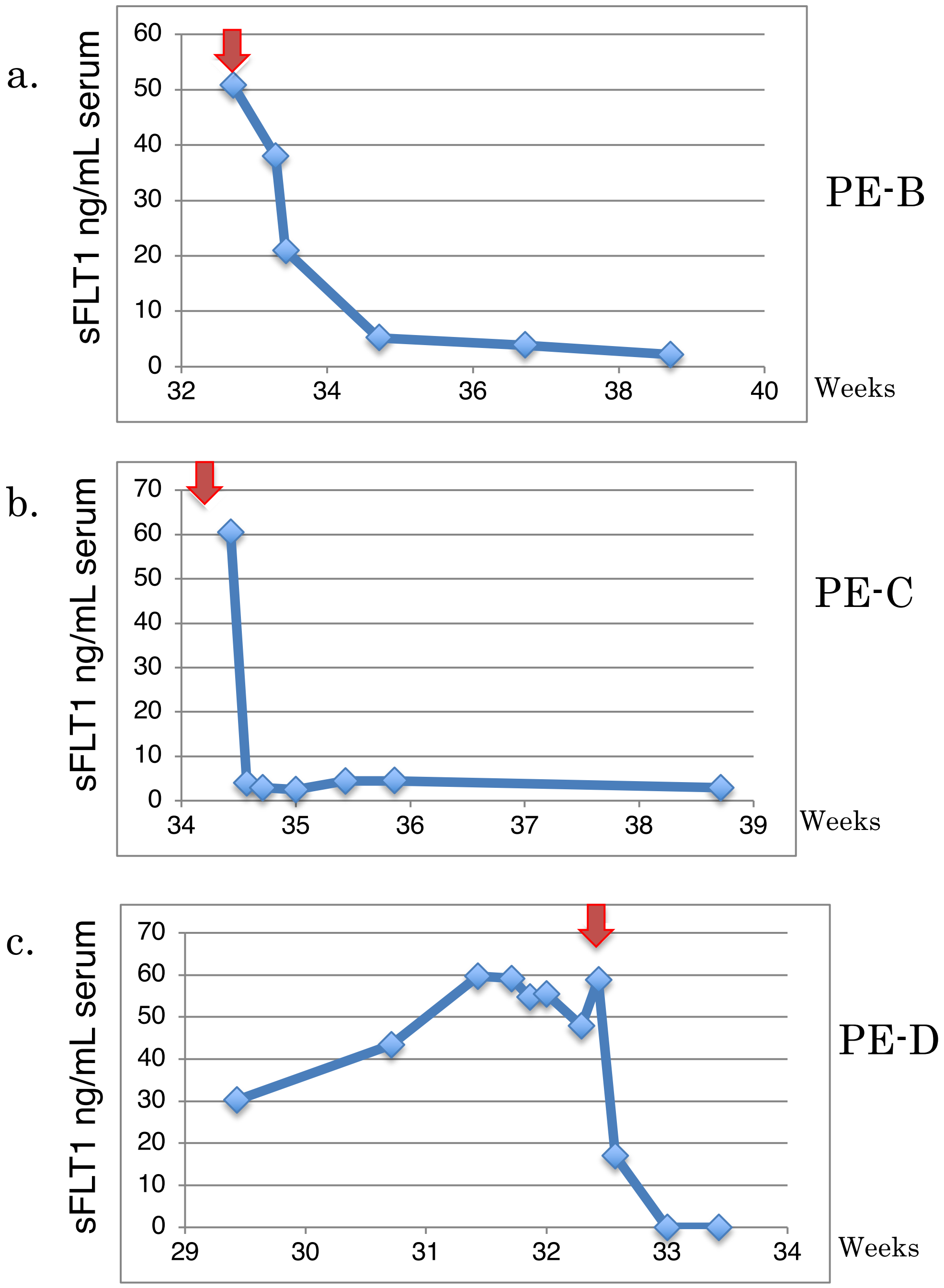

Supplementary Figure 1: The sFLT1 levels in the serum obtained from 3 severe PE cases (PE-B, C and D) at the stage of pre- and post-delivery. The values were detected with the HB-ELISA assay.
